# Crystal structure, spectroscopic characterization, and Hirshfeld surface analysis of (*E*)-6-(ferrocenylmethyl­idene)-6,7-di­hydro-5*H*-indeno­[5,6-*d*][1,3]dioxol-5-one

**DOI:** 10.1107/S2056989026002628

**Published:** 2026-03-24

**Authors:** José A. Méndez-Román, Alejandro Burgos-Suazo, Liz N. Santiago-Martoral, Dalice M. Piñero Cruz, Ingrid Montes-González

**Affiliations:** a Department of Chemistry University of Puerto Rico at Río Piedras San Juan Puerto Rico 00925-2537 USA; b University of Puerto Rico's Molecular Sciences Research Center San Juan Puerto Rico 00926 USA; Illinois State University, USA

**Keywords:** indanone, ferrocene, spectroscopic characaterization, crystal structure, Hirshfeld surface

## Abstract

The title compound, [Fe(C_5_H_5_)(C_16_H_11_O_3_)], was synthesized through a Claisen–Schmidt condensation. The title compound crystallizes in the monoclinic space group *P*2_1_/*c* with four mol­ecules per unit cell. In the crystal, mol­ecules are arranged in pairs with asymmetrical stacking by O⋯H inter­molecular inter­actions. The ferrocenyl indanone derivative is involved in several inter­molecular inter­actions, including C⋯H, C⋯O, C—H⋯O, and H⋯H contacts. The cyclo­penta­dienyl rings of ferrocene exhibit an average torsion angle of approximately −15.418°. The indanone fragment and the substituted cyclo­penta­dienyl ring are nearly coplanar, forming a dihedral angle of 8.18 (14)°. The Hirshfeld surface analysis qu­anti­fied the contributions from specific inter­actions involving the carbonyl moiety, π–π stacking, and H⋯H contacts. The two-dimensional fingerprint plots and NMR spectra were also analyzed.

## Abstract 2

A single crystal of ferrocenyl indanone, which crystallized in the monoclinic space group *P*2_1_/*c* was investigated by X-ray diffraction, Hirshfeld surface analysis and NMR spectroscopy. In the crystal, mol­ecules are arranged in pairs with asymmetrical stacking by O⋯H inter­molecular inter­actions.

## 1. Chemical context

Indanones or 2,3-di­hydro-1*H*-inden-1-ones are well-recognized pharmacophores due to their broad spectrum of biological activities, including anti­viral, anti­bacterial, anti­cancer, anti­malarial, anti-inflammatory, anti-Alzheimer, and cardiovascular properties (Turek *et al.*, 2017 ▸; Patil *et al.*, 2018 ▸). Several natural products bearing an indanone core have demonstrated significant bioactivity (Menezes, 2017 ▸). For example, natural products 1, 2, and 3 exhibit anti­bacterial, anti­spasmodic, and cytotoxic effects, underscoring the importance of the indanone scaffold in medicinal chemistry (Menezes, 2017 ▸). Given their promising anti­cancer potential, numerous indanone derivatives have been synthesized to explore structure–activity relationships and enhance therapeutic efficacy.
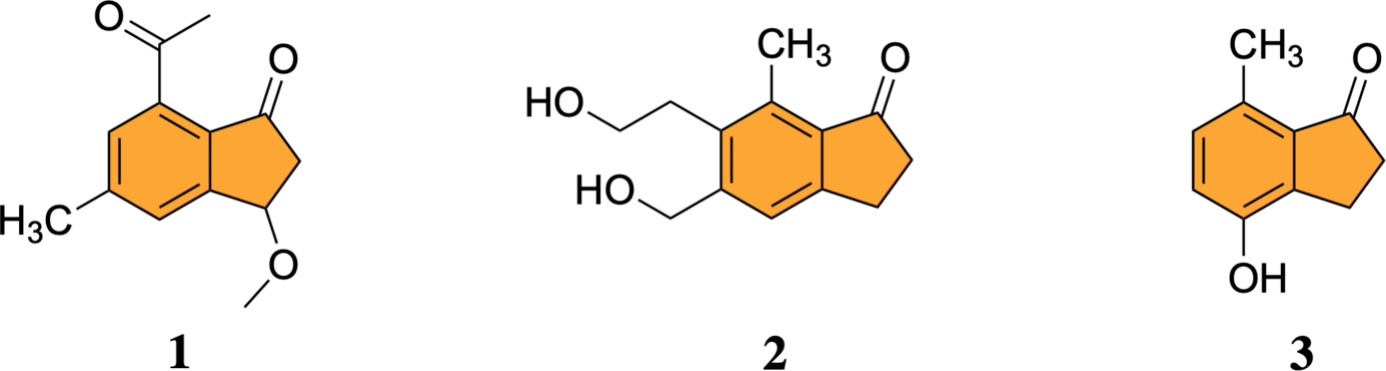


In parallel, organometallic compounds have garnered increasing inter­est over the past two decades for their diverse biological applications (Delgado-Rivera *et al.*, 2017 ▸). Among them, ferrocene – a metallocene compound – has been shown to enhance the biological activity of various pharmacophores (Zubair *et al.*, 2019 ▸). Owing to its chemical stability, high lipophilicity, and ability to improve anti­cancer, anti­malarial, and anti­bacterial activity, ferrocene has become a valuable building block in drug design (Zubair *et al.*, 2019 ▸; Kraatz *et al.*, 1997 ▸; Kealy & Pauson, 1951 ▸).

**Figure 1:**
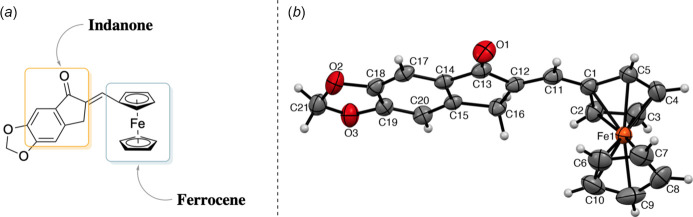
Representation of (*E*)-6-(ferrocenyl­methyl­idene)-6,7-di­hydro-5*H*-indeno­[5,6-*d*][1,3]dioxol-5-one. (a) Perspective drawing and (*b*) the mol­ecular structure with non-H atom numbering (displacement ellipsoids are drawn at the 50% probability level).

Ferrocen­yl–indanone frameworks have also been explored in fluorescence–electrochemical probe systems for analytical sensing (Song *et al.*, 2024 ▸; Tian *et al.*, 2024 ▸); however, the present study focuses on modifying the framework designed for target-oriented functionality rather than signal transduction. As a hybrid structure, the ferrocenyl-indanone scaffold may act through multiple mechanisms, combining the redox properties of ferrocene with inter­actions associated with the indanone pharmacophore. Herein, we report the crystal structure of a ferrocenyl-indanone hybrid synthesized as a potential bioactive mol­ecule (Fig. 1 ▸).
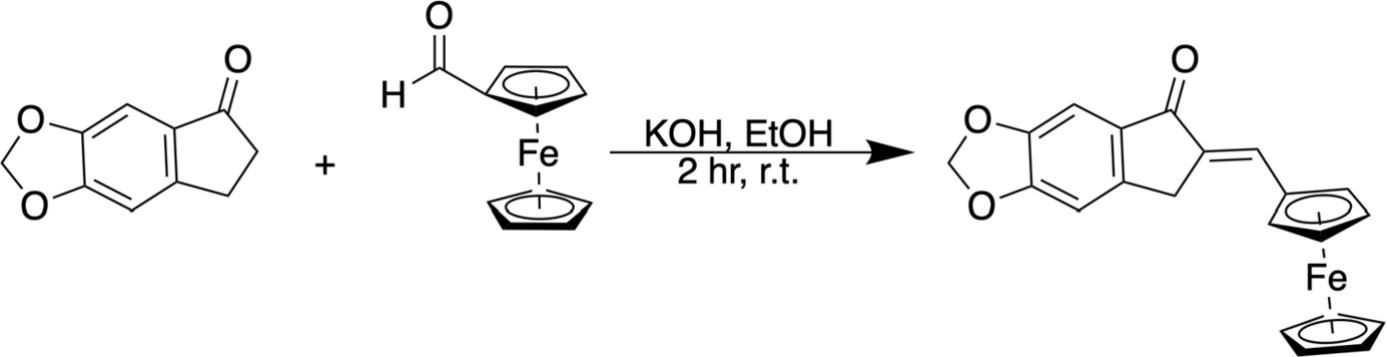


## 2. Structural commentary

**Figure 2:**
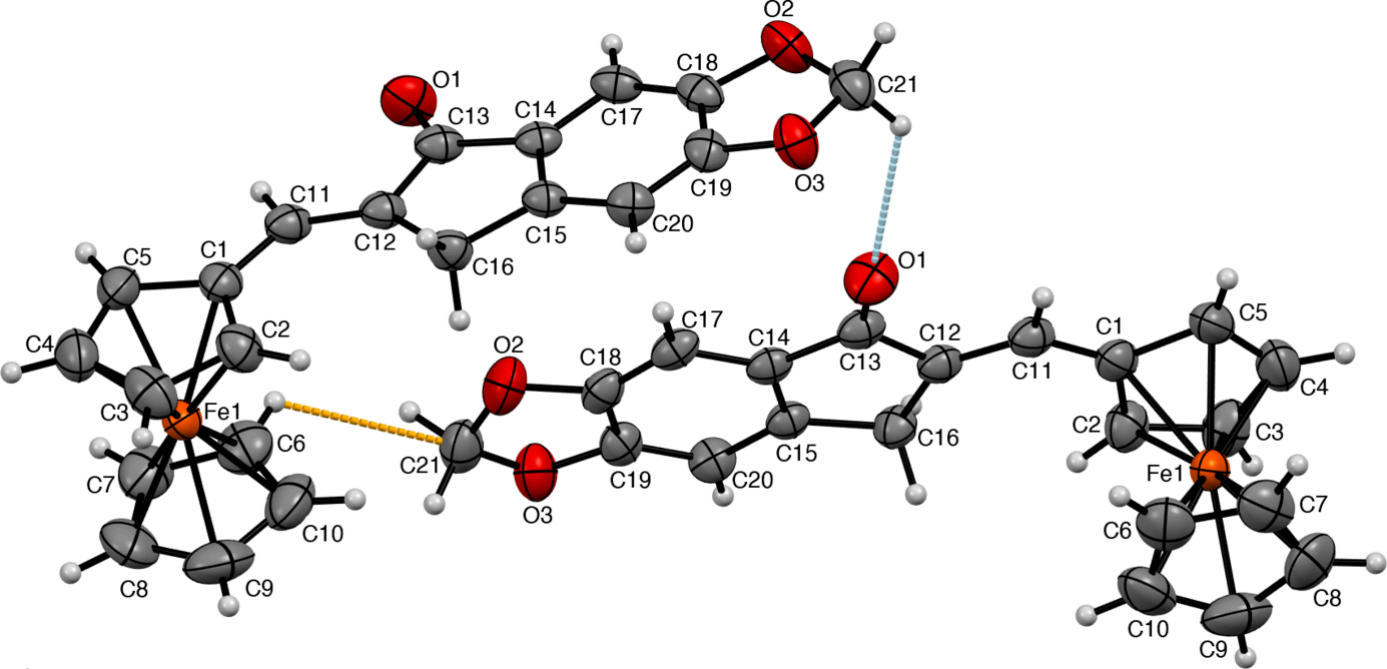
The title compound with the non-H atom numbering. The short contacts are shown as dashed orange (C⋯H inter­action) and blue (O⋯H inter­action) lines.

The title compound is a ferrocene–chalcone derivative with an extended conjugated system (Fig. 2 ▸). Two *sp*^3^-hybridized carbon atoms are incorporated within a cyclic framework, and their hydrogen atoms are oriented perpendicular to the indanone and cyclo­penta­dienyl cores. The aromatic C*sp*^2^—C*sp*^2^ bond lengths in the benzene ring are about 1.388 (3) Å, while the vinylic C11—C12 bond length of the alkene fragment is 1.333 (3) Å. The CH_2_ group of the cyclic ketone shows a C12—C16—C15 angle of 103.2 (1)°, whereas the corresponding 1,3-dioxol CH_2_ unit exhibits a O2—C21—O3 angle of 107.7 (2)°, consistent with their bonding environments.

The title compound crystallizes in the monoclinic space group *P*2_1_/*c* with one mol­ecule in the asymmetric unit. The Fe^II^ center is coordinated by two cyclo­penta­dienyl ligands, one of which bears a 6,7-di­hydro-5*H*-indeno­[5,6-*d*][1,3]dioxol-5-one substituent. The Fe—C distances lie in the range 2.033 (2)–2.048 (2) Å, with variations of less than 0.02 Å. The carbonyl oxygen participates in three short-contact inter­actions: C2—H2⋯O1, C21—H21*B*⋯O1, and C21⋯O1.

The cyclo­penta­dienyl rings exhibit an average torsion angle of approximately −15.418°, indicating a deviation from an ideal eclipsed *D*_5_*d* arrangement. The indanone fragment and the substituted cyclo­penta­dienyl ring are nearly coplanar, forming an inter­planar angle of 8.18 (14)°. Several inter­molecular contacts are present in the crystal, involving both the ferrocene and indanone moieties.

## 3. Supra­molecular features

**Figure 3:**
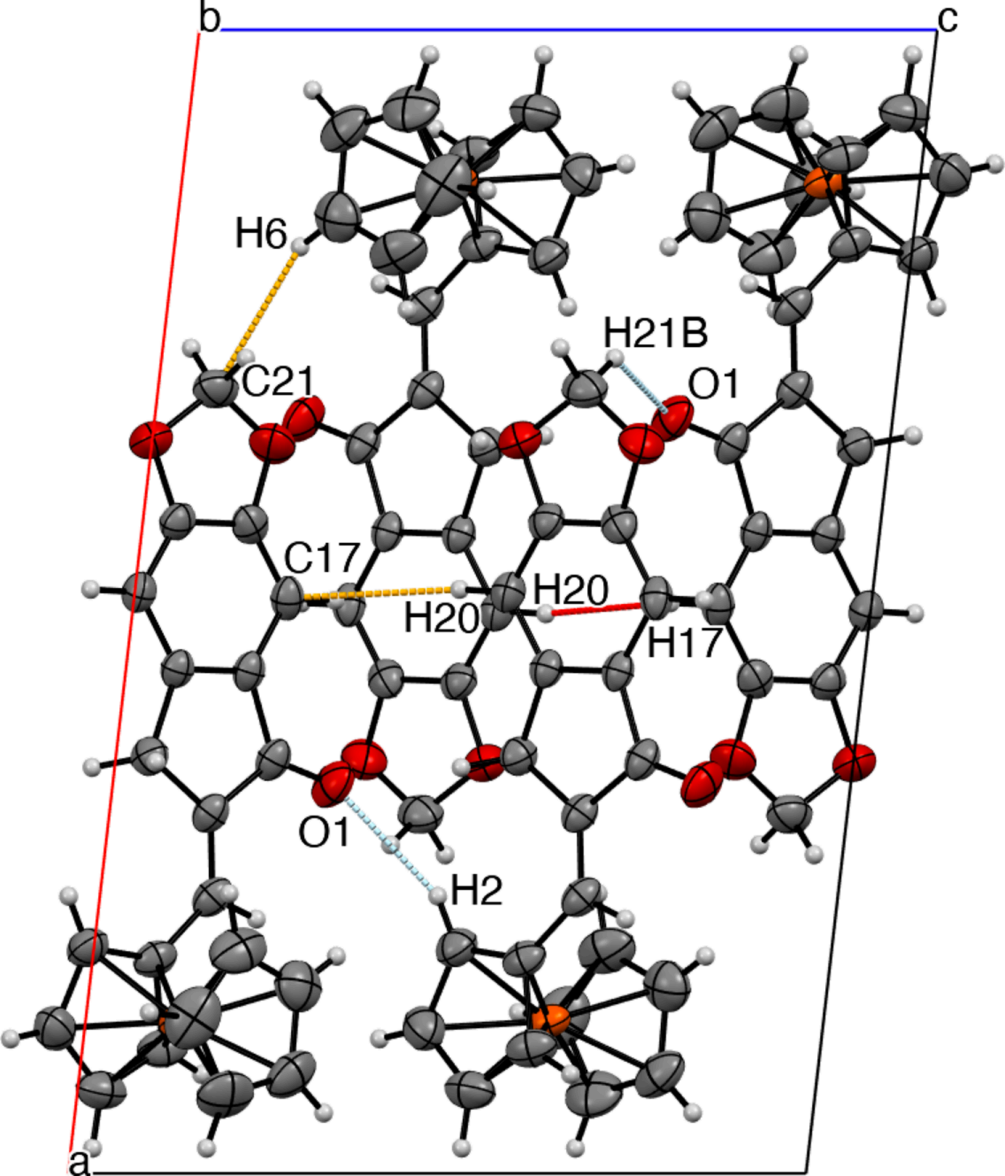
C⋯H (orange), C—H⋯O (blue), and H⋯H (red) short contact inter­actions viewed along the *b* axis.

The title ferrocenyl indanone derivative exhibits important inter­molecular inter­actions, namely three C⋯H, one C⋯O, two C—H⋯O, and one H⋯H inter­actions (Fig. 3 ▸, Table 1 ▸). The ferrocenyl moiety and the aromatic rings are perpendicular, and the molecular packing is arranged in layers extending along the *c*-axis direction. There are C—H⋯π inter­actions between a benzene ring’s C—H bond and a neighbouring benzene center of gravity with a C—H⋯*Cg* distance of 2.7662 (7) Å (the centroid was calculated by averaging the six carbon atoms that compose the ring). The CH_2_ group of the five-membered ketone ring exhibits C—H⋯O inter­actions with longer distances. The sheets are linked by out-of-plane C17⋯H20 and H17⋯H20 short contacts, generating stacks along the *c* axis. Also, there is an extension along the *b* axis where the ferrocene moieties are slightly perpendicular to each other, linked by C2—H2⋯O1 and C21—H21*B*⋯O1 contacts. The unit cell exhibits a twofold screw axis along [010] and a glide plane perpendicular to [010], resulting in a chain-fence-like crystal packing array.

## 4. Hirshfeld Surface Analysis

The Hirshfeld surface for the title compound was generated using the *CrystalExplorer21.5* software, and mapped over *d*_norm_, shape-index, and curvedness. The corresponding two-dimensional fingerprint plot analysis was also carried out (Spackman *et al.*, 2021 ▸; Spackman & Jayatilaka, 2009 ▸; Spackman & McKinnon, 2002 ▸).

**Figure 4:**
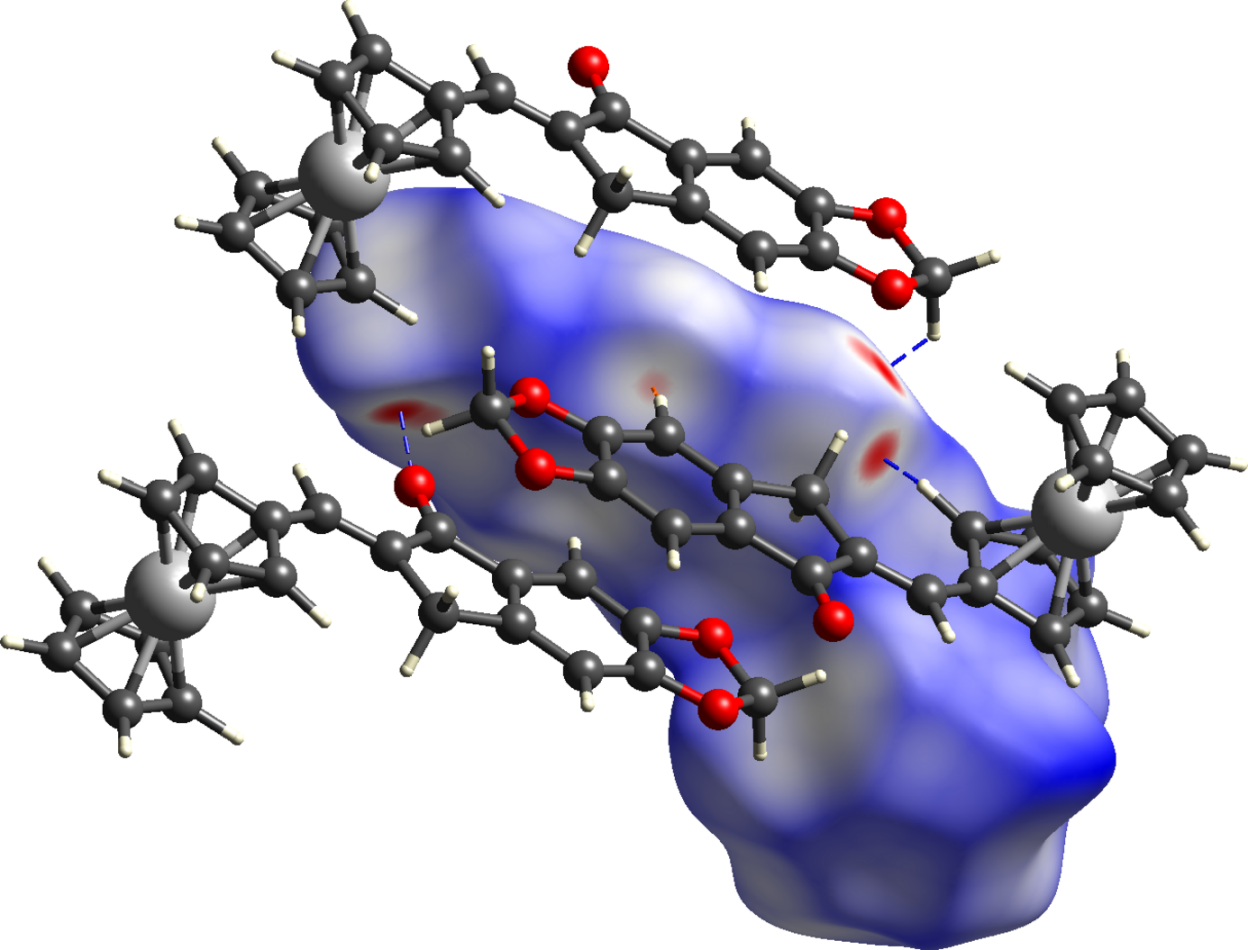
Hirshfeld surface evaluated over *d_norm_* for the title compound with adjacent mol­ecules and short contacts (H⋯O/O⋯H blue and C⋯H/H⋯C orange).

The generated surface evaluated over *d*_norm_ (−0.2096–1.2772 a.u.) shows several red spots, mostly distributed over the indanone moiety, indicating the short contacts within the crystal packing (Fig. 4 ▸). The bright-red spots, having the shortest distances, are principally for H⋯O/O⋯H inter­actions. Contacts of C⋯H/H⋯C type are also underlined in the surface, but the red spots are of lighter intensity (*i.e.*, longer distances). Likewise, there are many light blue/white colored spots, some of which are representative of H⋯H and C⋯C inter­molecular inter­actions. It should be emphasized that even though H⋯H inter­actions are displayed as light blue/white regions, this inter­action accounts for half the total inter­molecular contacts in the crystal packing, evidenced by the two-dimensional fingerprint plots (Fig. 7).

**Figure 5:**
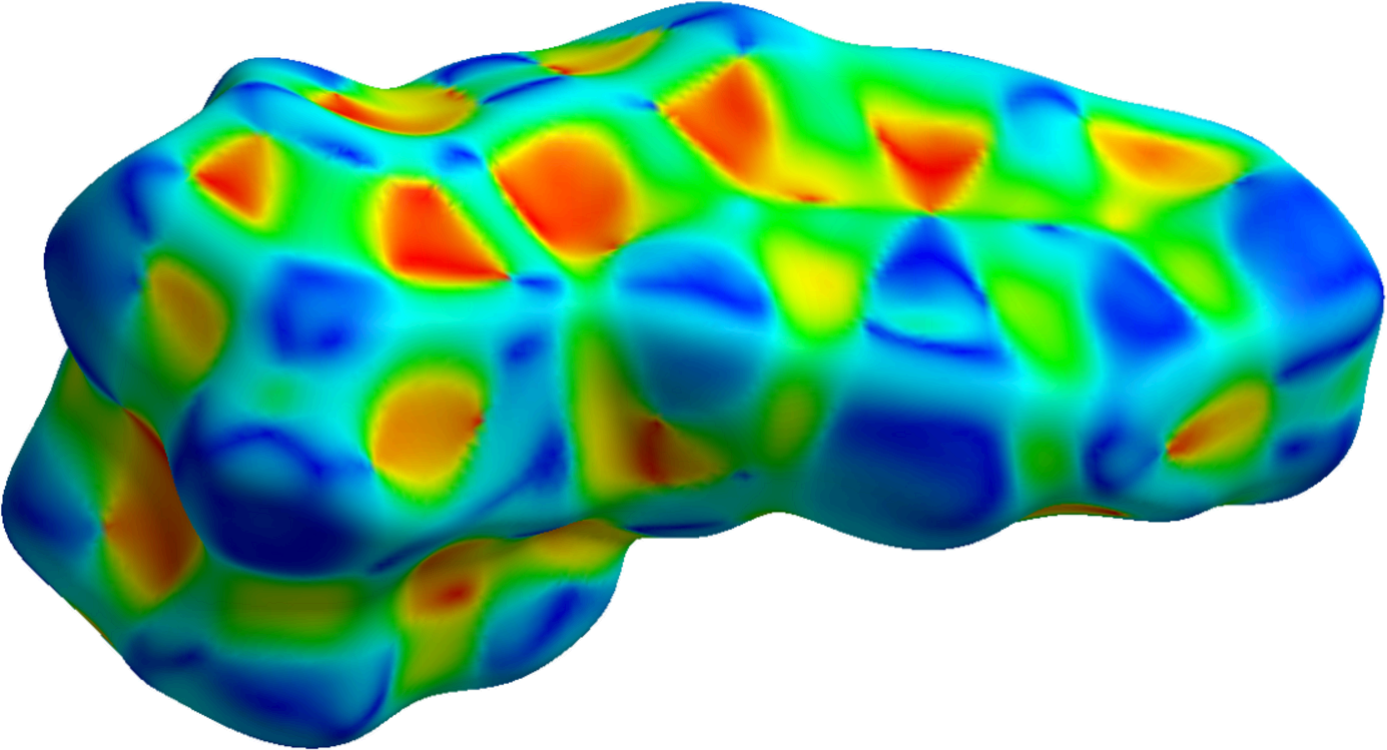
Hirshfeld surface mapped over shape-index for the title compound.

The shape-index surface (Fig. 5 ▸) reveals π–π stacking, indicated by adjacent red–yellow and blue–green triangles, located primarily on the indanone aromatic ring and substituted ferrocene ring regions. Furthermore, the observed pattern of hollows and bumps delineates mol­ecular inter­locking that gives way to crystallization.

**Figure 6:**
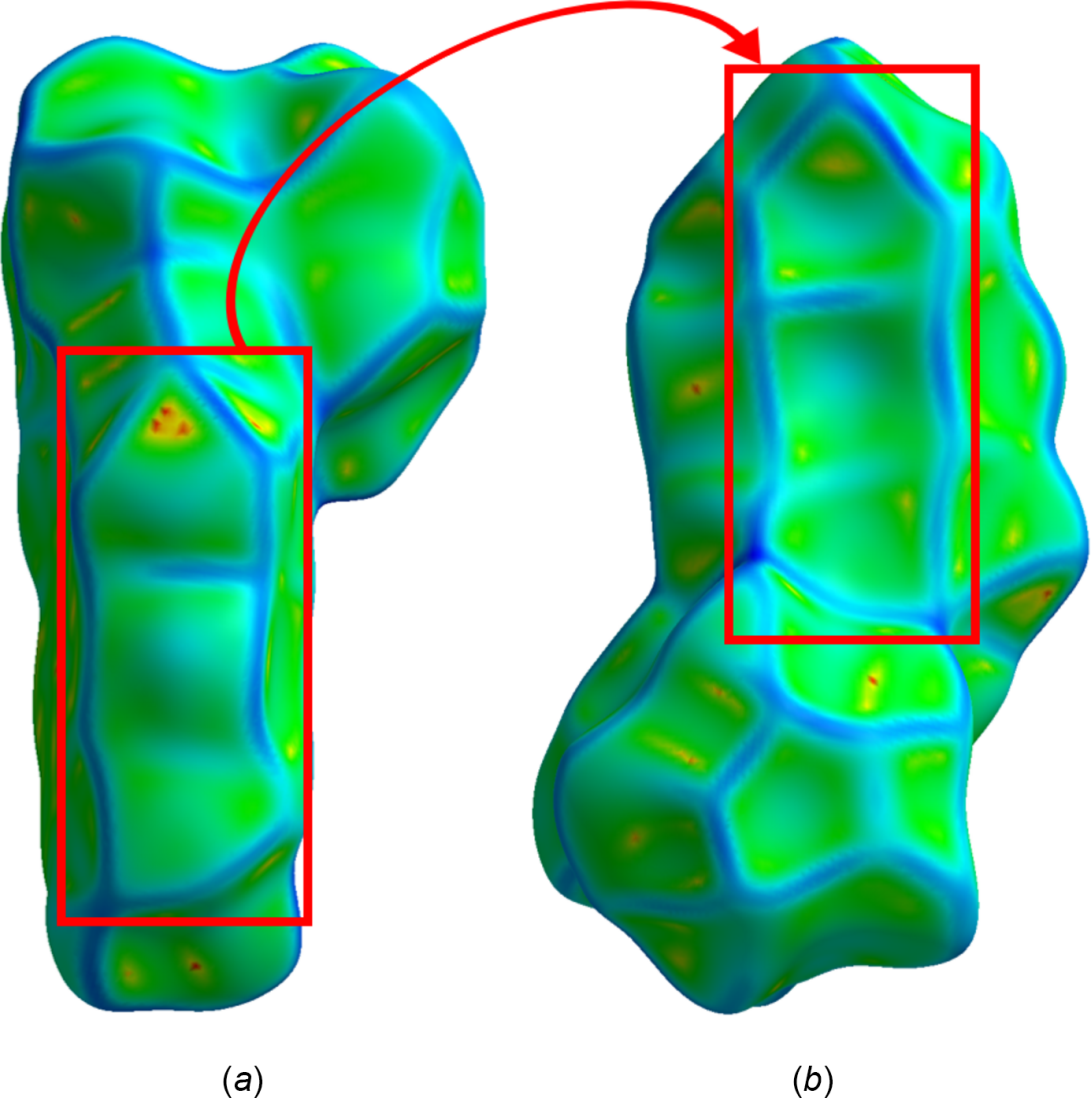
Hirshfeld surface evaluated over curvedness for the title compound, viewed from the side (*a*) and bottom (*b*) of the mol­ecule.

The curvedness feature is shown in Fig. 6 ▸, where a combination of flat segments and positive curvatures are seen. In the left part of Fig. 6 ▸*a*, a completely green region is observed, resulting from the planar stacking of mol­ecules, specifically for the indanone scaffold. Moreover, the red box in Fig. 6 ▸*a*, when turned over, perfectly matches the red box in Fig. 6 ▸*b*, outlining the inter­locking of neighboring mol­ecules.

**Figure 7:**
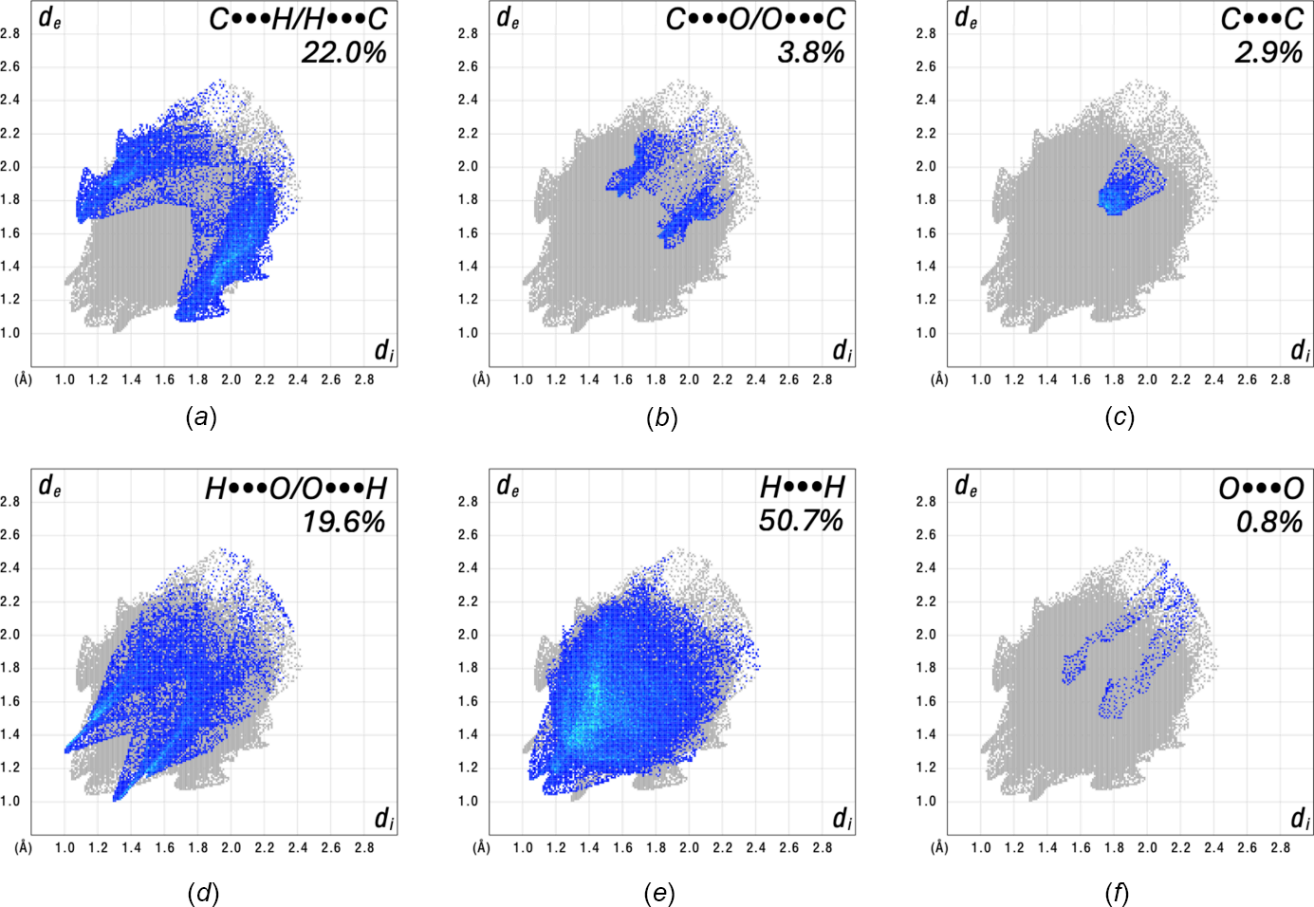
Fingerprint plots showing the inter­molecular inter­actions present in the crystal structure.

The two-dimensional fingerprint plots (Fig. 7 ▸) are symmetric and include characteristic features for sets of inter­actions. The C⋯H/H⋯C (22.0%) contact plot contains two pairs of wings, representative of C—H⋯π inter­actions. H⋯O/O⋯H (19.6%) type contacts occur over a long range of distances and present a pair of peaks characteristic of hydrogen bonding. The H⋯H (50.7%) contacts, which contribute half of all inter­actions, also cover a broad range of distances and include a large number of points around ∼1.4 Å (*d*_i_ = *d*_e_), showing the importance of this short contact for the crystal packing. These last three inter­actions account for more than 92% of all contacts, whereas C⋯O/O⋯C (3.8%), O⋯O (0.8%), and C⋯C (2.9%) make much smaller percentage contributions to the crystal packing. However, the C⋯C fingerprint plot exhibits a large concentration of inter­actions around 1.8 Å (*d*_i_ = *d*_e_), denoting π–π stacking.

## 5. Database survey

A search of the Cambridge Structural Database (CSD, Version 6.01, November 2025 update; Groom *et al.*, 2016 ▸; ConQuest Version 2025.3.1; Build 470021) for structures containing a ferrocen­yl–indanone fragment returned 17 hits. Closely related structures include POGMOR (Song *et al.*, 2024 ▸), POGMUX (Song *et al.*, 2024 ▸), and WOZVAM (Tian *et al.*, 2024 ▸), which differ only by *R*-group substituents. POGMOR (*R* = OCOCH_3_) and POGMUX (*R* = OH) crystallize in the space group *P*2_1_/*c* like the title compound, with angles β = 94.025 (2) and 103.646 (1)°, respectively, compared to β = 96.553 (1)° for the title compound; WOZVAM (*R* = OCOOCH_2_CH=CH_2_) crystallizes in the *P*ī space group.

For all these structures, the substituents seem to influence the crystal packing with respect to the benzene ring. In the title compound, the heterocyclic substituent promotes an extended planar arrangement that enables π–π stacking between parallel benzene rings. Conversely, in POGMOR, the benzene ring exhibits π–π and C—H⋯π inter­actions with the ferrocene unit of a neighbouring mol­ecule. In POGMUX, the benzene ring exhibits π–π inter­actions with the chalcone alkene of an adjacent mol­ecule. In WOZVAM, neighbouring indanone cores are oppositely oriented, allowing π–π contacts involving benzene-ring carbon atoms.

The inter-planar angles between the substituted cyclo­penta­dienyl ring and the indanone core differ among structures (title compound = 8.18°; POGMOR = 8.08°; POGMUX = 2.95°; WOZVAM = 17.68°). Differences are also observed in the torsion angles between the ferrocene cyclo­penta­dienyl rings, where the title compound exhibits the highest deviation from an eclipsed conformation (title compound = −15.418°; POGMOR = 9.148°; POGMUX = −8.808°; WOZVAM = −6.028°).

## 6. Synthesis and crystallization

All reagents were obtained commercially and used without further purification. 6,7-Di­hydro-5*H*-indeno­[5,6-*d*][1,3]dioxol-5-one (0.25 mmol, 1.0 equiv.) was dissolved in ethanol (6 mL), and a solution of KOH (0.50 mmol) in ethanol was added dropwise. The mixture was stirred for 5 min at room temperature before adding ferrocenyl-carboxaldehyde (0.25 mmol, 1.0 equiv.). The reaction mixture was stirred at room temperature for 2 h, during which a red–orange precipitate formed. The solid was collected by filtration and washed with cold water. Recrystallization was performed with a mixture of acetone and water to purify the compound and obtain single crystals. As a result, a red–orange compound was obtained with an 85% yield. NMR analyses were performed on a Bruker AV-500 spectrometer using deuterated dimethyl sulfoxide as solvent (DMSO-*d*_6_). The solvent signals at δ 2.50 and 3.30 ppm were used as inter­nal standards for proton and δ 40.0 ppm for carbon. ^1^H-NMR (500 MHz, DMSO-*d*_6_) δ 3.74 (*s*, 2H), 4.20 (*s*, 5H), 4.56 (*t*, 2H), 4.75 (*t*, 2H), 6.18 (*s*, 2H), 7.15 (*s*, 1H), 7.16 (*s*, 1H) 7.31 (*s*, 1H). ^13^C-NMR (125 MHz, DMSO-*d*_6_) δ 32.0, 70.0, 71.2, 71.7, 78.8, 102.4, 102.8, 106.5, 132.6, 133.2, 133.6, 147.1, 148.5, 153.8, 190.7.

## 7. Refinement

Crystal data, data collection and structure refinement details are summarized in Table 2 ▸. H atoms were treated by a mixture of independent and constrained refinement. H16*A* and H16*B* were located in a difference-Fourier map and refined with independent coordinates and isotropic displacement parameters. All other H atoms (secondary C21(H21*A*, H21*B*) and aromatic hydrogens) were treated using a constrained riding model with fixed C—H distances and *U*_iso_(H) = 1.2*U*_eq_(C).

## Supplementary Material

Crystal structure: contains datablock(s) I. DOI: 10.1107/S2056989026002628/ej2018sup1.cif

Structure factors: contains datablock(s) I. DOI: 10.1107/S2056989026002628/ej2018Isup2.hkl

CCDC reference: 2536986

Additional supporting information:  crystallographic information; 3D view; checkCIF report

## Figures and Tables

**Table 1:** Selected H⋯C/C⋯H, C⋯O/O⋯C, C—H⋯O, and H⋯H short-contact inter­actions (Å)

C21⋯H6^i^	2.86	C2—H2⋯O1^iv^	2.50
C21⋯O1^ii^	3.053 (3)	C17⋯H20^v^	2.85
C21—H21B⋯O1^ii^	2.38	H17⋯H20^v^	2.40
C11⋯H21A^iii^	2.87		

Symmetry codes: (i) 1 − *x*, 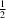 + *y*, 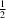 − *z*; (ii) 1 − *x*, −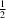 + *y*, 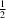 − *z*; (iii) 1 − *x*, 2 − *y*, 1 − *z*; (iv) *x*, 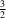 − *y*, 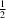 + *z*; (v) *x*, 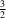 − *y*, −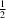 + *z*.

**Table 2:** Experimental details

Crystal data	
Chemical formula	[Fe(C_5_H_5_)(C_16_H_11_O_3_)]
*M*_r_	372.19
Crystal system, space group	Monoclinic, *P*2_1_/*c*
Temperature (K)	300
*a*, *b*, *c* (Å)	18.2411 (2), 7.5076 (1), 11.7029 (1)
β (°)	96.553 (1)
*V* (Å^3^)	1592.21 (3)
*Z*	4
Radiation type	Cu *K*α
μ (mm^−1^)	7.74
Crystal size (mm)	0.30 × 0.10 × 0.03

Data collection	
Diffractometer	SuperNova, Single source at offset/far, HyPix3000
Absorption correction	Multi-scan (*CrysAlis PRO*; Rigaku OD, 2021 ▸)
*T*_min_, *T*_max_	0.430, 0.811
No. of measured, independent and observed [*I* > 2σ(*I*)] reflections	26037, 2903, 2669
*R*_int_	0.052
(sin θ/λ)_max_ (Å^−1^)	0.603

Refinement	
*R*[*F*^2^ > 2σ(*F*^2^)], *wR*(*F*^2^), *S*	0.030, 0.077, 1.03
No. of reflections	2903
No. of parameters	235
H-atom treatment	H atoms treated by a mixture of independent and constrained refinement
Δρ_max_, Δρ_min_ (e Å^−3^)	0.19, −0.23

Computer programs: *CrysAlis PRO* (Rigaku OD, 2021 ▸), *SHELXT2014/5* (Sheldrick, 2015*a* ▸), *SHELXL2018/3* (Sheldrick, 2015*b* ▸) and *OLEX2* (Dolomanov *et al.*, 2009 ▸).

## supplementary crystallographic information

### (E)-6-(Ferrocenylmethylidene)-6,7-dihydro-5H-indeno[5,6-d][1,3]dioxol-5-one Crystal data

**Table:**

[Fe(C_5_H_5_)(C_16_H_11_O_3_)]	*F*(000) = 768
*M**_r_* = 372.19	*D*_x_ = 1.553 Mg m^−^^3^
Monoclinic, *P*2_1_/*c*	Cu *K*α radiation, λ = 1.54184 Å
*a* = 18.2411 (2) Å	Cell parameters from 14416 reflections
*b* = 7.5076 (1) Å	θ = 2.4–68.3°
*c* = 11.7029 (1) Å	µ = 7.74 mm^−^^1^
β = 96.553 (1)°	*T* = 300 K
*V* = 1592.21 (3) Å^3^	Block, clear reddish orange
*Z* = 4	0.30 × 0.10 × 0.03 mm

### (E)-6-(Ferrocenylmethylidene)-6,7-dihydro-5H-indeno[5,6-d][1,3]dioxol-5-one Data collection

**Table:**

SuperNova, Single source at offset/far, HyPix3000 diffractometer	2903 independent reflections
Radiation source: micro-focus sealed X-ray tube, SuperNova (Cu) X-ray Source	2669 reflections with *I* > 2σ(*I*)
Mirror monochromator	*R*_int_ = 0.052
Detector resolution: 10.0000 pixels mm^-1^	θ_max_ = 68.3°, θ_min_ = 2.4°
ω scans	*h* = −21→21
Absorption correction: multi-scan (CrysAlisPro; Rigaku OD, 2021)	*k* = −7→8
*T*_min_ = 0.430, *T*_max_ = 0.811	*l* = −14→14
26037 measured reflections	

### (E)-6-(Ferrocenylmethylidene)-6,7-dihydro-5H-indeno[5,6-d][1,3]dioxol-5-one Refinement

**Table:**

Refinement on *F*^2^	Hydrogen site location: mixed
Least-squares matrix: full	H atoms treated by a mixture of independent and constrained refinement
*R*[*F*^2^ > 2σ(*F*^2^)] = 0.030	*w* = 1/[σ^2^(*F*_o_^2^) + (0.0388*P*)^2^ + 0.4964*P*] where *P* = (*F*_o_^2^ + 2*F*_c_^2^)/3
*wR*(*F*^2^) = 0.077	(Δ/σ)_max_ = 0.001
*S* = 1.03	Δρ_max_ = 0.19 e Å^−^^3^
2903 reflections	Δρ_min_ = −0.23 e Å^−^^3^
235 parameters	Extinction correction: SHELXL-2018/3 (Sheldrick 2015b), Fc^*^=kFc[1+0.001xFc^2^λ^3^/sin(2θ)]^-1/4^
0 restraints	Extinction coefficient: 0.00065 (12)
Primary atom site location: dual	

### (E)-6-(Ferrocenylmethylidene)-6,7-dihydro-5H-indeno[5,6-d][1,3]dioxol-5-one Special details

**Table:**

Geometry. All esds (except the esd in the dihedral angle between two l.s. planes) are estimated using the full covariance matrix. The cell esds are taken into account individually in the estimation of esds in distances, angles and torsion angles; correlations between esds in cell parameters are only used when they are defined by crystal symmetry. An approximate (isotropic) treatment of cell esds is used for estimating esds involving l.s. planes.

### (E)-6-(Ferrocenylmethylidene)-6,7-dihydro-5H-indeno[5,6-d][1,3]dioxol-5-one Fractional atomic coordinates and isotropic or equivalent isotropic displacement parameters (Å^2^)

**Table:**

	*x*	*y*	*z*	*U*_iso_*/*U*_eq_	
Fe1	0.13324 (2)	0.51816 (4)	0.37179 (3)	0.03779 (12)	
O1	0.34011 (9)	0.9984 (2)	0.20148 (12)	0.0539 (4)	
O2	0.63811 (8)	0.9482 (2)	0.33674 (13)	0.0541 (4)	
O3	0.64122 (7)	0.7776 (2)	0.50157 (12)	0.0505 (4)	
C1	0.18754 (10)	0.7508 (3)	0.41518 (16)	0.0403 (4)	
C2	0.19744 (11)	0.6262 (3)	0.50846 (17)	0.0462 (5)	
H2	0.242327	0.581661	0.542240	0.055*	
C3	0.12680 (12)	0.5827 (4)	0.54034 (19)	0.0552 (6)	
H3	0.117584	0.505036	0.598942	0.066*	
C4	0.07282 (12)	0.6772 (3)	0.4681 (2)	0.0548 (6)	
H4	0.022098	0.672265	0.470739	0.066*	
C5	0.10932 (11)	0.7802 (3)	0.39128 (19)	0.0482 (5)	
H5	0.086600	0.854931	0.334498	0.058*	
C6	0.16370 (14)	0.4412 (4)	0.2175 (2)	0.0619 (6)	
H6	0.189860	0.510392	0.170188	0.074*	
C7	0.08655 (13)	0.4338 (4)	0.2136 (2)	0.0617 (6)	
H7	0.052880	0.496339	0.163045	0.074*	
C8	0.06914 (14)	0.3154 (4)	0.2993 (2)	0.0682 (7)	
H8	0.021998	0.286451	0.316285	0.082*	
C9	0.13596 (16)	0.2485 (3)	0.3550 (2)	0.0735 (8)	
H9	0.140583	0.166440	0.414985	0.088*	
C10	0.19470 (14)	0.3268 (3)	0.3045 (2)	0.0671 (7)	
H10	0.244749	0.306452	0.325195	0.081*	
C11	0.24163 (10)	0.8275 (3)	0.34745 (16)	0.0392 (4)	
H11	0.222317	0.894894	0.284415	0.047*	
C12	0.31498 (10)	0.8150 (2)	0.36279 (15)	0.0356 (4)	
C13	0.36171 (10)	0.9098 (3)	0.28627 (14)	0.0372 (4)	
C14	0.43898 (9)	0.8793 (2)	0.33291 (13)	0.0328 (4)	
C15	0.44114 (9)	0.7728 (2)	0.43030 (14)	0.0329 (4)	
C16	0.36477 (10)	0.7180 (3)	0.45437 (16)	0.0380 (4)	
H16A	0.3597 (11)	0.589 (3)	0.4451 (17)	0.042 (5)*	
H16B	0.3549 (10)	0.749 (3)	0.5306 (18)	0.042 (5)*	
C17	0.50295 (11)	0.9472 (2)	0.29216 (15)	0.0376 (4)	
H17	0.501179	1.019449	0.227362	0.045*	
C18	0.56760 (10)	0.8997 (3)	0.35413 (15)	0.0383 (4)	
C19	0.56998 (10)	0.7958 (3)	0.45294 (15)	0.0375 (4)	
C20	0.50818 (9)	0.7297 (3)	0.49407 (14)	0.0383 (4)	
H20	0.510681	0.660452	0.560287	0.046*	
C21	0.68600 (11)	0.8550 (3)	0.42183 (18)	0.0505 (5)	
H21A	0.721497	0.936643	0.461401	0.061*	
H21B	0.712730	0.762747	0.385922	0.061*	

### (E)-6-(Ferrocenylmethylidene)-6,7-dihydro-5H-indeno[5,6-d][1,3]dioxol-5-one Atomic displacement parameters (Å^2^)

**Table:**

	*U*^11^	*U*^22^	*U*^33^	*U*^12^	*U*^13^	*U*^23^
Fe1	0.03119 (17)	0.03412 (19)	0.04630 (19)	−0.00120 (12)	−0.00313 (12)	−0.00276 (12)
O1	0.0580 (9)	0.0560 (10)	0.0440 (8)	−0.0040 (7)	−0.0100 (7)	0.0183 (7)
O2	0.0456 (8)	0.0617 (10)	0.0561 (8)	−0.0160 (7)	0.0098 (7)	0.0019 (7)
O3	0.0370 (7)	0.0660 (10)	0.0471 (7)	−0.0043 (7)	−0.0020 (6)	−0.0002 (7)
C1	0.0372 (9)	0.0347 (10)	0.0469 (10)	−0.0016 (8)	−0.0042 (8)	−0.0072 (8)
C2	0.0402 (10)	0.0516 (13)	0.0448 (10)	−0.0053 (9)	−0.0041 (8)	−0.0021 (9)
C3	0.0531 (12)	0.0653 (15)	0.0481 (11)	−0.0121 (11)	0.0094 (9)	−0.0053 (11)
C4	0.0381 (10)	0.0572 (14)	0.0697 (14)	0.0000 (10)	0.0095 (9)	−0.0140 (11)
C5	0.0393 (10)	0.0375 (11)	0.0661 (13)	0.0047 (9)	−0.0011 (9)	−0.0072 (10)
C6	0.0684 (15)	0.0585 (15)	0.0598 (14)	0.0014 (13)	0.0119 (12)	−0.0167 (12)
C7	0.0614 (14)	0.0594 (15)	0.0588 (13)	0.0046 (12)	−0.0165 (11)	−0.0176 (12)
C8	0.0587 (14)	0.0595 (16)	0.0836 (18)	−0.0218 (12)	−0.0043 (12)	−0.0177 (14)
C9	0.102 (2)	0.0341 (13)	0.0796 (17)	−0.0004 (13)	−0.0090 (15)	−0.0036 (12)
C10	0.0582 (14)	0.0564 (15)	0.0838 (17)	0.0187 (12)	−0.0047 (12)	−0.0223 (13)
C11	0.0425 (10)	0.0318 (10)	0.0406 (9)	−0.0015 (8)	−0.0071 (8)	−0.0012 (8)
C12	0.0409 (10)	0.0289 (9)	0.0352 (9)	−0.0031 (8)	−0.0030 (7)	−0.0018 (7)
C13	0.0458 (10)	0.0311 (10)	0.0325 (9)	−0.0036 (8)	−0.0045 (7)	−0.0017 (8)
C14	0.0425 (9)	0.0275 (9)	0.0275 (8)	−0.0024 (7)	−0.0002 (7)	−0.0040 (7)
C15	0.0388 (9)	0.0290 (9)	0.0301 (8)	−0.0030 (7)	0.0008 (7)	−0.0025 (7)
C16	0.0389 (10)	0.0366 (11)	0.0379 (10)	−0.0021 (8)	0.0022 (8)	0.0054 (8)
C17	0.0507 (11)	0.0327 (10)	0.0294 (8)	−0.0078 (8)	0.0042 (8)	−0.0013 (7)
C18	0.0426 (10)	0.0363 (11)	0.0365 (9)	−0.0097 (8)	0.0067 (7)	−0.0072 (8)
C19	0.0373 (9)	0.0400 (11)	0.0340 (9)	−0.0024 (8)	−0.0016 (7)	−0.0080 (8)
C20	0.0442 (10)	0.0397 (11)	0.0303 (9)	−0.0010 (8)	0.0016 (7)	0.0021 (7)
C21	0.0399 (10)	0.0564 (14)	0.0552 (12)	−0.0092 (10)	0.0056 (9)	−0.0115 (10)

### (E)-6-(Ferrocenylmethylidene)-6,7-dihydro-5H-indeno[5,6-d][1,3]dioxol-5-one Geometric parameters (Å, º)

**Table:**

Fe1—C1	2.0435 (19)	C6—C10	1.401 (4)
Fe1—C2	2.0397 (19)	C7—H7	0.9300
Fe1—C3	2.048 (2)	C7—C8	1.404 (4)
Fe1—C4	2.048 (2)	C8—H8	0.9300
Fe1—C5	2.034 (2)	C8—C9	1.408 (4)
Fe1—C6	2.033 (2)	C9—H9	0.9300
Fe1—C7	2.048 (2)	C9—C10	1.410 (4)
Fe1—C8	2.044 (2)	C10—H10	0.9300
Fe1—C9	2.035 (3)	C11—H11	0.9300
Fe1—C10	2.035 (2)	C11—C12	1.333 (3)
O1—C13	1.222 (2)	C12—C13	1.486 (3)
O2—C18	1.374 (2)	C12—C16	1.510 (2)
O2—C21	1.430 (3)	C13—C14	1.470 (2)
O3—C19	1.365 (2)	C14—C15	1.389 (2)
O3—C21	1.432 (2)	C14—C17	1.406 (2)
C1—C2	1.433 (3)	C15—C16	1.510 (2)
C1—C5	1.439 (3)	C15—C20	1.396 (2)
C1—C11	1.453 (3)	C16—H16A	0.98 (2)
C2—H2	0.9300	C16—H16B	0.96 (2)
C2—C3	1.420 (3)	C17—H17	0.9300
C3—H3	0.9300	C17—C18	1.359 (3)
C3—C4	1.414 (3)	C18—C19	1.392 (3)
C4—H4	0.9300	C19—C20	1.368 (3)
C4—C5	1.409 (3)	C20—H20	0.9300
C5—H5	0.9300	C21—H21A	0.9700
C6—H6	0.9300	C21—H21B	0.9700
C6—C7	1.404 (3)		
			
H6···C21	2.256		
			
C1—Fe1—C3	68.76 (9)	C1—C5—H5	125.6
C1—Fe1—C4	68.95 (8)	C4—C5—Fe1	70.34 (13)
C1—Fe1—C7	128.96 (10)	C4—C5—C1	108.78 (19)
C1—Fe1—C8	167.94 (10)	C4—C5—H5	125.6
C2—Fe1—C1	41.10 (8)	Fe1—C6—H6	125.5
C2—Fe1—C3	40.66 (8)	C7—C6—Fe1	70.47 (14)
C2—Fe1—C4	68.50 (9)	C7—C6—H6	125.7
C2—Fe1—C7	166.96 (10)	C10—C6—Fe1	69.93 (14)
C2—Fe1—C8	150.66 (10)	C10—C6—H6	125.7
C3—Fe1—C4	40.38 (9)	C10—C6—C7	108.6 (2)
C3—Fe1—C7	151.69 (10)	Fe1—C7—H7	126.5
C4—Fe1—C7	118.90 (10)	C6—C7—Fe1	69.29 (13)
C5—Fe1—C1	41.34 (7)	C6—C7—H7	126.0
C5—Fe1—C2	68.81 (9)	C8—C7—Fe1	69.77 (13)
C5—Fe1—C3	68.06 (10)	C8—C7—C6	108.0 (2)
C5—Fe1—C4	40.39 (9)	C8—C7—H7	126.0
C5—Fe1—C7	109.16 (10)	Fe1—C8—H8	125.8
C5—Fe1—C8	130.32 (10)	C7—C8—Fe1	70.10 (13)
C5—Fe1—C9	168.83 (10)	C7—C8—H8	126.2
C5—Fe1—C10	149.43 (11)	C7—C8—C9	107.6 (2)
C6—Fe1—C1	106.86 (9)	C9—C8—Fe1	69.49 (14)
C6—Fe1—C2	128.25 (9)	C9—C8—H8	126.2
C6—Fe1—C3	167.06 (10)	Fe1—C9—H9	126.0
C6—Fe1—C4	150.85 (10)	C8—C9—Fe1	70.13 (14)
C6—Fe1—C5	117.30 (10)	C8—C9—H9	125.8
C6—Fe1—C7	40.24 (10)	C8—C9—C10	108.4 (2)
C6—Fe1—C8	67.73 (11)	C10—C9—Fe1	69.71 (14)
C6—Fe1—C9	67.63 (12)	C10—C9—H9	125.8
C6—Fe1—C10	40.29 (10)	Fe1—C10—H10	125.7
C8—Fe1—C3	118.87 (10)	C6—C10—Fe1	69.78 (13)
C8—Fe1—C4	110.13 (10)	C6—C10—C9	107.3 (2)
C8—Fe1—C7	40.12 (10)	C6—C10—H10	126.3
C9—Fe1—C1	149.08 (10)	C9—C10—Fe1	69.75 (14)
C9—Fe1—C2	116.95 (10)	C9—C10—H10	126.3
C9—Fe1—C3	109.45 (11)	C1—C11—H11	115.4
C9—Fe1—C4	130.81 (11)	C12—C11—C1	129.22 (17)
C9—Fe1—C7	67.51 (11)	C12—C11—H11	115.4
C9—Fe1—C8	40.38 (10)	C11—C12—C13	121.40 (16)
C10—Fe1—C1	115.26 (10)	C11—C12—C16	129.99 (17)
C10—Fe1—C2	106.96 (9)	C13—C12—C16	108.57 (15)
C10—Fe1—C3	129.38 (11)	O1—C13—C12	126.58 (17)
C10—Fe1—C4	168.31 (11)	O1—C13—C14	126.35 (18)
C10—Fe1—C7	67.83 (10)	C14—C13—C12	107.06 (14)
C10—Fe1—C8	68.18 (11)	C15—C14—C13	109.28 (15)
C10—Fe1—C9	40.54 (11)	C15—C14—C17	122.70 (16)
C18—O2—C21	105.97 (15)	C17—C14—C13	128.00 (16)
C19—O3—C21	105.93 (15)	C14—C15—C16	111.69 (15)
C2—C1—Fe1	69.31 (11)	C14—C15—C20	120.87 (16)
C2—C1—C5	106.50 (18)	C20—C15—C16	127.43 (16)
C2—C1—C11	129.76 (17)	C12—C16—H16A	110.9 (12)
C5—C1—Fe1	68.97 (11)	C12—C16—H16B	112.3 (12)
C5—C1—C11	123.57 (18)	C15—C16—C12	103.25 (15)
C11—C1—Fe1	122.89 (13)	C15—C16—H16A	109.0 (12)
Fe1—C2—H2	126.1	C15—C16—H16B	112.4 (12)
C1—C2—Fe1	69.59 (10)	H16A—C16—H16B	108.8 (17)
C1—C2—H2	125.9	C14—C17—H17	122.4
C3—C2—Fe1	69.97 (12)	C18—C17—C14	115.29 (16)
C3—C2—C1	108.13 (18)	C18—C17—H17	122.4
C3—C2—H2	125.9	O2—C18—C19	109.30 (16)
Fe1—C3—H3	126.7	C17—C18—O2	128.41 (18)
C2—C3—Fe1	69.38 (12)	C17—C18—C19	122.22 (17)
C2—C3—H3	125.7	O3—C19—C18	109.92 (16)
C4—C3—Fe1	69.82 (13)	O3—C19—C20	126.91 (17)
C4—C3—C2	108.6 (2)	C20—C19—C18	123.14 (17)
C4—C3—H3	125.7	C15—C20—H20	122.1
Fe1—C4—H4	126.5	C19—C20—C15	115.75 (16)
C3—C4—Fe1	69.80 (13)	C19—C20—H20	122.1
C3—C4—H4	126.0	O2—C21—O3	107.69 (15)
C5—C4—Fe1	69.27 (12)	O2—C21—H21A	110.2
C5—C4—C3	108.03 (18)	O2—C21—H21B	110.2
C5—C4—H4	126.0	O3—C21—H21A	110.2
Fe1—C5—H5	125.9	O3—C21—H21B	110.2
C1—C5—Fe1	69.69 (11)	H21A—C21—H21B	108.5
			
Fe1—C1—C2—C3	−59.58 (15)	C8—C9—C10—C6	−0.3 (3)
Fe1—C1—C5—C4	59.68 (15)	C10—C6—C7—Fe1	59.74 (17)
Fe1—C1—C11—C12	−97.1 (2)	C10—C6—C7—C8	0.5 (3)
Fe1—C2—C3—C4	−58.99 (16)	C11—C1—C2—Fe1	−116.2 (2)
Fe1—C3—C4—C5	−58.90 (15)	C11—C1—C2—C3	−175.74 (19)
Fe1—C4—C5—C1	−59.28 (14)	C11—C1—C5—Fe1	116.30 (18)
Fe1—C6—C7—C8	−59.22 (17)	C11—C1—C5—C4	175.98 (18)
Fe1—C6—C10—C9	59.93 (17)	C11—C12—C13—O1	−3.7 (3)
Fe1—C7—C8—C9	−59.62 (17)	C11—C12—C13—C14	175.37 (17)
Fe1—C8—C9—C10	−59.40 (18)	C11—C12—C16—C15	−174.19 (19)
Fe1—C9—C10—C6	−59.95 (16)	C12—C13—C14—C15	0.5 (2)
O1—C13—C14—C15	179.56 (18)	C12—C13—C14—C17	−177.68 (17)
O1—C13—C14—C17	1.4 (3)	C13—C12—C16—C15	3.6 (2)
O2—C18—C19—O3	0.9 (2)	C13—C14—C15—C16	1.9 (2)
O2—C18—C19—C20	178.81 (18)	C13—C14—C15—C20	−177.39 (16)
O3—C19—C20—C15	177.61 (17)	C13—C14—C17—C18	178.63 (17)
C1—C2—C3—Fe1	59.34 (14)	C14—C15—C16—C12	−3.5 (2)
C1—C2—C3—C4	0.3 (3)	C14—C15—C20—C19	−1.2 (3)
C1—C11—C12—C13	−177.50 (18)	C14—C17—C18—O2	−178.51 (18)
C1—C11—C12—C16	0.1 (3)	C14—C17—C18—C19	−1.9 (3)
C2—C1—C5—Fe1	−59.43 (14)	C15—C14—C17—C18	0.7 (3)
C2—C1—C5—C4	0.3 (2)	C16—C12—C13—O1	178.25 (19)
C2—C1—C11—C12	−7.6 (3)	C16—C12—C13—C14	−2.7 (2)
C2—C3—C4—Fe1	58.72 (16)	C16—C15—C20—C19	179.56 (18)
C2—C3—C4—C5	−0.2 (3)	C17—C14—C15—C16	−179.78 (16)
C3—C4—C5—Fe1	59.23 (16)	C17—C14—C15—C20	0.9 (3)
C3—C4—C5—C1	0.0 (2)	C17—C18—C19—O3	−176.33 (17)
C5—C1—C2—Fe1	59.21 (13)	C17—C18—C19—C20	1.6 (3)
C5—C1—C2—C3	−0.4 (2)	C18—O2—C21—O3	−10.4 (2)
C5—C1—C11—C12	177.7 (2)	C18—C19—C20—C15	0.0 (3)
C6—C7—C8—Fe1	58.92 (17)	C19—O3—C21—O2	10.9 (2)
C6—C7—C8—C9	−0.7 (3)	C20—C15—C16—C12	175.81 (18)
C7—C6—C10—Fe1	−60.08 (17)	C21—O2—C18—C17	−177.1 (2)
C7—C6—C10—C9	−0.1 (3)	C21—O2—C18—C19	6.0 (2)
C7—C8—C9—Fe1	60.01 (17)	C21—O3—C19—C18	−7.3 (2)
C7—C8—C9—C10	0.6 (3)	C21—O3—C19—C20	174.85 (19)
C8—C9—C10—Fe1	59.66 (18)		
